# Follow up Imaging Protocols after Endovascular Aneurysm Repair: Results of the International FOREVAR Survey

**DOI:** 10.1016/j.ejvsvf.2025.07.005

**Published:** 2025-07-28

**Authors:** Bich L. Tran, Sabrina L.M. Zwetsloot, Martin Teraa, Fabien Lareyre, Leszek Kukulski, Vincent Jongkind, European Vascular Research Collaborative, Alexandra Gratl, Florian Enzmann, Gert J. de Borst, Lewis Meecham, Stefano Ancetti, Paolo Spath, Albert Busch, Bergrós Jóhannesdóttir, Alexander Gombert, Mohammad E. Barbati, Panagiotis Doukas, Katariina Noronen, Robert Hinchliffe, Alexandru Predenciuc, Qasam Ghulam, Angelos Karelis, Maram Darwish, Cecilie Markvard Møller, Matt Spreadbury, Willemien van de Water, Desiree van den Hondel, Alexander Croo, Gilles Uijtterhaegen, Ryan Gouveia e Melo, Vaiva Dabravolskaite, Christian Zielasek, Salome Weiss, Vishal Amlani, Aoife Kiernan, Petar Zlatanovic, Nikolaos Patelis, Max Hoebink, Harm Ebben, Kak Khee Yeung

**Affiliations:** g Department of Vascular Surgery, Medical University of Innsbruck, Innsbruck, Austria; h Department of Vascular Surgery, University Medical Center Utrecht, the Netherlands; i Department of Vascular Surgery, University Hospital Wales, Cardiff, UK; j Department of Vascular Surgery, Bologna University Hospital, Bologna, Italy; k Division of Vascular and Endovascular Surgery, Department of Visceral, Thoracic and Vascular Surgery, Technical University of Dresden and University Hospital Carl-Gustav Carus, Dresden, Germany; l Department of Vascular Surgery, Bordeaux University Hospital, Bordeaux, France; m Clinic of Vascular and Endovascular Surgery, RWTH Aachen University Hospital, Aachen, Germany; n Department of Vascular Surgery, Helsinki University Hospital, Helsinki, Finland; o Department of Vascular Surgery, Bristol Medical School, University of Bristol, United Kingdom; p Division of Vascular Surgery, Institute of Emergency Medicine, Nicolae Testemitanu State University of Medicine and Pharmacy, Chisinau, the Republic of Moldova; q Department of Vascular Surgery, Capio Privathospital, Hellerup, Denmark; r Vascular Center, Department of Thoracic Surgery and Vascular Diseases, Skåne University Hospital, Malmö, Sweden; s Health Education England, Vascular and Endovascular Surgery, East Midlands Deanery, Nottingham, United Kingdom; t Department of Cardiothoracic and Vascular Surgery, Aarhus University Hospital, Denmark; u Department of Vascular Surgery, Oslo University Hospital, Oslo, Norway; v Department of Vascular Surgery, Maastricht University Medical Center, Maastricht, the Netherlands; w Department of Vascular Surgery, Frisius MC, Leeuwarden, the Netherlands; x Department of Vascular Surgery, AZORG Hospital, Aalst, Belgium; y Department of Vascular Surgery, AZ Sint-Lucas, Brugge, Belgium; z Vascular Surgery Department, Hospital Santa Maria, Unidade Local Saúde de Santa Maria, Portugal; aa Faculdade de Medicina da Universidade de Lisboa, Centro Cardiovascular da Universidade de Lisboa (CCUL@RISE), Portugal; ab Department of Vascular Surgery, Inselspital, Bern University Hospital, Bern, Switzerland; ac Department of Vascular Surgery, Sahlgrenska University Hospital, Gothenburg, Sweden; ad Department of Vascular Surgery, Bon Secours Health System, Dublin, Ireland; ae Clinic for Vascular and Endovascular Surgery, University Clinical Centre of Serbia, Belgrade, Serbia; af Department of Vascular Surgery, Mouwasat Hospital, Saudi Arabia; ag Department of Surgery, Amsterdam University Medical Center, Amsterdam, the Netherlands; a Department of Surgery, Amsterdam UMC, Vrije Universiteit Amsterdam, Amsterdam, the Netherlands; b Amsterdam Cardiovascular Sciences, Atherosclerosis & Ischaemic Syndromes, Amsterdam, the Netherlands; c Department of Surgery, Amsterdam University Medical Centres, University of Amsterdam, Amsterdam, the Netherlands; d Department of Vascular Surgery, University Medical Centre Utrecht, Utrecht, the Netherlands; e Department of Vascular Surgery, Hospital of Antibes Juan-les-Pins, Université Côte d'Azur, Nice, France; f Department of Vascular, General and Transplantation Surgery, Wroclaw Medical University, Wrocław, Poland

**Keywords:** Endovascular aortic repair, Follow up imaging, Protocol, Survey, Vascular surgery

## Abstract

**Objective:**

Endovascular repair for aortic aneurysms necessitates routine follow up due to potential late complications, such as endograft occlusions, endoleaks, and late aneurysm rupture. Guidelines recommend periodic post-operative imaging, including computed tomography angiography (CTA) or duplex ultrasound, to monitor aneurysm status and stent integrity. The efficacy of these follow up protocols is controversial, with concerns about increased healthcare costs and patient morbidity. This survey aimed to assess global variance in follow up imaging protocols among vascular surgeons, interventional radiologists, and vascular surgery trainees.

**Methods:**

A global English web based survey was conducted over nine months and distributed through email, social media, and direct messaging to vascular surgeons, interventional radiologists, and other vascular specialists. Procedure specific questions included imaging techniques used and follow up duration.

**Results:**

The FOREVAR Survey was completed by 693 respondents from 65 countries. Most participants reported having a standardised follow up imaging protocol following all, or nearly all, elective endovascular aneurysm repairs (EVAR) in their centre (98%). The follow up protocols displayed substantial variation. In addition to completion angiography, other imaging was often performed before discharge (cone beam computed tomography 11%, CTA 27%, or DUS 17%). CTA is most often performed at first follow up (63%), while DUS is most frequently used during later follow up (56%). Median intervals to follow up imaging were eight weeks (first), 39 weeks (second), and 52 weeks (third). Follow up never ceased in 51% of cases. Comparable results were reported for complex EVAR (cEVAR) and thoracic EVAR (TEVAR), but with a greater proportion of patients receiving CTA after cEVAR at long term follow up (74%). Magnetic resonance imaging was used by a minority in the follow up of EVAR, cEVAR, and TEVAR (4%, 1%, and 4%, respectively).

**Conclusions:**

This global survey showed substantial variation in follow up imaging protocols after EVAR, cEVAR, and TEVAR. Most of these protocols lead to long term radiation exposure during follow up.

## HIGHLIGHTS

• There was great global variation in follow up after endovascular aortic repair.
• Countries differed in the type and frequency of imaging used.
• There was variation in length of follow up and median interval to follow up imaging.
• Complex and thoracic repair frequently had similar protocols to standard repair.
• There was a lack of consensus on which imaging follow up protocol was considered optimal.

## INTRODUCTION

Endovascular aneurysm repair (EVAR) has evoked a revolution in the treatment of aortic aneurysms (AA), not only for infrarenal, but also for paravisceral, thoraco-abdominal, and thoracic aneurysms. EVAR is related to specific long term complications, such as graft occlusions, endoleaks, and post-EVAR aneurysm rupture. Therefore, long term follow up is deemed necessary.^1^ Initial post-operative clinical and imaging evaluations following EVAR are imperative to determine procedural efficacy. The primary objective of the initial follow up is to evaluate patient recovery, identify peri-procedural complications, and confirm the effective exclusion of the aneurysm. Follow up imaging may be performed by computed tomography angiography (CTA), duplex ultrasound (DUS) with or without contrast enhancement, X ray, magnetic resonance imaging (MRI), or a combination. Each technique has its own advantages and disadvantages.

Current stent graft instructions for use (IFU) include recommendations regarding regular follow up with up to three CT scans during the first post-operative year, and continued regularly after that.^2^^,^^3^ Imaging is used to assess aneurysm exclusion, presence of endoleaks, adequate sealing, kinking, compression, side branch patency in complex EVAR (cEVAR), and monitor progression of residual aortic pathology.^1^^,^^2^ There is very limited evidence for annual imaging after EVAR and while routine surveillance occasionally identifies significant findings requiring re-intervention, adverse events occur despite this surveillance.^1^^,^^2^ The value of follow up imaging after EVAR is debatable, as routine imaging follow up can also have negative effects on patients, including morbidity and death from corrective interventions and the use of radiation.^1^^,^^2^ Furthermore, it increases healthcare costs. According to the recent European Society for Vascular Surgery (ESVS) guidelines on treatment of AA, defining the optimal follow up after EVAR is one of the key issues.^1^

In the absence of prospective studies on the value of follow up, imaging follow up protocols may vary significantly between different centres and, to date, no consensus has been reached on a standard follow up imaging protocol.^4^ The primary aim of this survey was to study the variation of follow up imaging protocols after standard, complex (fenestrated and branched EVAR), and thoracic EVAR (TEVAR) amongst vascular surgeons, interventional radiologists, and vascular surgery trainees in different centres and countries around the world.

## METHODS

### Study design

A worldwide survey was conducted in collaboration with the European Vascular Research Collaborative (EVRC, eurovascresearch.com). A web based survey was conducted from September 2022 until June 2023. The survey was created in English and dispersed through email, social media promotion, and direct messaging.

### Survey construction

The survey was constructed and deployed using the web based survey platform SurveyMonkey (SurveyMonkey Inc.®, San Mateo, CA, USA, surveymonkey.com). General information was collected regarding hospital and country of practice, type of specialty, years of experience in the clinical setting, and the use of standard protocols for imaging follow up after EVAR, cEVAR, and TEVAR. Features were incorporated into the survey design to allow participants to bypass questions if they were irrelevant based on previously provided answers or if the standard EVAR protocols were identical to the cEVAR or TEVAR protocols. Procedure specific information was collected: details on imaging techniques used, time until follow up, and information about long term follow up. The complete survey is displayed in the Supplementary material.

### Survey deployment

Participants could complete the FOllow up afteR EndoVascular Aneurysm Repair (FOREVAR) Survey from September 2022 until June 2023. The survey was accessible via a web based link or QR code. Survey invitations were repeatedly disseminated by EVRC group members via social media, email, and during scientific conferences. The survey was also accessible via the EVRC website. Invitations were also shared by national vascular surgical societies. Weekly updates were sent to the EVRC group through email. The General Data Protection Regulation (GDPR) was employed when handling data of participating vascular specialists and respondents. IP restriction was implemented, allowing participants to only complete one survey per IP address, and contact information was asked of participants to ensure reliable data collection.

### Statistical analysis

SPSS version 28 (IBM Corp., Armonk, NY, USA) was used for data analyses. Before data analysis, double entry data were removed manually based on contact details. Descriptive statistical analyses were performed. The Kruskal Wallis tests or independent *t* tests were performed to analyse potential differences in the types of imaging modality used during follow up and between the different types of endovascular techniques. In addition, the differences between medical and or vascular centres worldwide were identified.

## RESULTS

### Baseline characteristics and general information

The FOREVAR survey was completed by respondents from 65 countries (Table 1). In total, 452 respondents (65%) filled in their centre and at least 62 centres where two or more respondents derived from were identified. Mean completion time of the survey was 11 minutes. Following the removal of duplicates and incorrect entries through a thorough manual selection process, 693 valid responses were retained for analysis. Most respondents were vascular surgeons (78%); others included interventional radiologists (13%) and vascular surgery trainees (7%). Among participants, the most common duration of clinical experience ranged 6–10 years (36%), which corresponded to their experience in performing EVAR.

**Table 1:** Baseline characteristics of respondent to the FOREVAR Survey.

Characteristics	*n* (%)
*Specialty*	
Vascular surgeon	538 (78)
Interventional radiologist	92 (13)
Vascular surgery trainee or resident	52 (7)
Other	11 (2)
*Continent working*	
Europe	469 (68)
North America	57 (8)
South America	21 (3)
Africa	41 (6)
Asia	73 (10)
Australia/Oceania	32 (5)
*Age – y*	
25-34	162 (23)
35-44	273 (39)
45-54	138 (20)
55-64	64 (10)
65+	9 (1)
Not answered	47 (7)
*Status*	
Consultant	418 (60)
Resident	186 (27)
Other	42 (6)
Not answered	47 (7)
*Clinical experience – y*	
>20	107 (15)
11-20	126 (18)
6-10	247 (36)
1-5	157 (23)
Not answered	56 (8)
*Experience in endovascular aortic procedures – y*	
>20	60 (9)
11-20	141 (20)
6-10	250 (36)
1-5	186 (27)
Not answered	56 (8)
*What kind of practice are you working at?*	
University hospital	295 (43)
Private hospital	236 (34)
State hospital	96 (113)
Other (please specify)	11 (2)
Not answered	55 (8)

Data are presented as *n* (%).

### Standard EVAR

Follow up protocols after standard EVAR are reported in Table 2. Most participants had a standardised follow up imaging protocol in their centre for all patients after standard elective infrarenal EVAR (*n* = 448, 67%); this was 100% among vascular surgeons. A total of 205 participants (31%) had a standardised post-operative protocol for at least 80% of the patients, while 18 respondents had no standard protocol (3%). A distinction was made between adhering to protocol in 100% of patients and in >80% of patients because realistically protocols are not followed in all cases. A high variance was reported for these follow up protocols. In total, 44% of respondents only performed a completion angiogram before discharge, while many performed either an intra-operative cone beam computed tomography (CBCT), post-operative CTA or DUS. At first imaging follow up, CTA was used most frequently (63%), followed by DUS (40%). A minority used MRI, while abdominal Xray (AXR) was used by 12%. Of those who did not perform CTA at first follow up, 15% of respondents performed a CTA or CBCT before discharge. Thus, 78% of respondents were consistent with the current European Society for Vascular Surgery (ESVS) guideline recommendation to perform early CTA after EVAR.^1^ At longer term follow up, DUS was most frequently used (56%), while CTA was performed by 39% of respondents. The median interval between intervention and first imaging follow up was 8 weeks (interquartile range [IQR] 4, 44). This was 39 weeks for the second follow up (IQR 24, 52), and 52 weeks for third follow up (IQR 37, 69). Most imaging follow up appointments took place at the same medical centre in which the EVAR procedure was performed (78%). Respondents continued lifelong imaging follow up after EVAR in 51%, while other respondents ended follow up imaging after a certain number of years (13%) or when the patient had reached a certain age (30%). If the procedure was within IFU and the imaging showed no endoleak (including type 2), 98% of respondents still performed standard follow up.

**Table 2:** Follow up protocols after standard endovascular aortic repair (EVAR), complex EVAR, and thoracic EVAR.

Variable	EVAR (*n* = 693)	cEVAR (*n* = 133)	TEVAR (*n* = 172)
*Standardised protocol*			
Yes	67	49	53
Yes for 80%	31	33	35
No	3	18	13
*What imaging before discharge*∗			
None	15	6	13
Completion angiography only	44	30	38
Cone beam CT during hybrid procedure	11	13	8
CTA	27	50	42
DUS/CEUS	17	11	6
*What imaging first follow up*∗			
CTA	63	83	90
DUS/CEUS	40	23	10
Xray	12	3	1
MRI/MRA	4	1	4
*What imaging second follow up*∗			
CTA	39	74	91
DUS/CEUS	56	33	9
Xray	18	5	2
MRI/MRA	5	1	2
*What imaging third follow up*∗			
CTA	40	86	91
DUS/CEUS	61	17	5
Xray	16	6	5
MRI/MRA	10	1	5
*How many weeks after elective is imaging follow up*∗			
1st performed	8 [4, 44]	6 [4, 12]	6 [4, 12]
2^nd^ follow up weeks	39 [24, 52]	30 [24, 52]	48 [24, 52]
3^rd^ follow up weeks	52 [37, 69]	52 [51.75, 100]	52 [48, 100]
*Where does follow up take place*∗			
Referring centre	18		18
Performing centre	78		73
Other	4		9
*When does follow up end*∗			
After a certain age of the patient	30	9	9
After a certain number of years	13	5	6
Never	51	79	76
Other	5	7	9

Data are presented as *n* (%) or median (interquartile range [IQR]). CT = computed tomography; CTA = computed tomography angiography; CEUS = contrast enhanced ultrasound; DUS = duplex ultrasound; MRI = magnetic resonance imaging; MRA = magnetic resonance angiography.

∗ Multiple answer options were possible for selecting imaging modalities.

### cEVAR

cEVAR procedures, including fenestrated, branched, and chimney EVAR, were performed by 548 respondents, representing 79% of all respondents (Table 2). Of the 133 respondents who noted their cEVAR protocol, 82% performed CTA at first imaging follow up. Of those who did not perform CTA at first follow up, 9% of respondents performed CTA and one performed CBCT before discharge; thus, 92% of respondents were consistent with current ESVS guidelines.^1^ For the first follow up after cEVAR, 23% of respondents use DUS, its use increasing during subsequent follow up appointments. The first imaging follow up took place within a median of six weeks (IQR 4, 12). The second follow up imaging occurred at a median of 30 weeks (IQR 24, 52), and the third at a median of 52 weeks (IQR 51.75, 100). Respondents reported discontinuing follow up imaging after a certain number of years in 5%, when the patient had reached a certain age in 9%, or when the patient was deemed unfit for re-intervention (other, 7%).

### TEVAR

TEVAR procedures were performed by 512 participants, accounting for 74% of respondents (Table 2). After TEVAR, the most performed imaging was a completion angiogram, conducted by 44% of the respondents, while 10% used CBCT and 32% performed a CTA before discharging the patient. Of these, 172 respondents filled in their TEVAR protocol; 88% performed CTA at first imaging follow up. Of those who did not perform CTA at first follow up, 4% of respondents performed CTA. Thus, 93% of respondents were consistent with current ESVS guidelines.^5^ On average, the first follow up imaging appointment after TEVAR occurred within a median of six weeks (IQR 4, 12). The second follow up imaging was typically performed at a median of 48 weeks (IQR 24, 52), and the third at a median of 52 weeks (IQR 48, 100).

## DISCUSSION

The FOREVAR survey provides a comprehensive overview of the current follow up imaging protocols after standard infrarenal EVAR, cEVAR, and TEVAR, highlighting significant variability in post-EVAR monitoring approaches. Most respondents used a completion angiogram as the final imaging step, but many also performed CTA or CBCT before discharging the patient. CTA was the most frequently used imaging modality for the first follow up, while DUS was commonly used in subsequent evaluations. For longer term imaging follow up, CTA or DUS was used in 94% of participants.^1^ Nearly three quarters of the respondents reported the same follow up protocol for cEVAR and TEVAR as for standard EVAR. This substantial variability underscores the absence of consensus on the optimal imaging follow up protocol. There is a critical need for evidence on the best type, schedule, and duration of follow up imaging and its cost effectiveness for specific aortic procedures.

Most respondents adhered to ESVS guideline recommendations.^1^^,^^5^ CTA was the most frequently used first imaging modality following EVAR, cEVAR, and TEVAR. If CTA was not the first imaging modality, intra-operative CBCT or CTA before discharge were often performed. In total, 22% of respondents’ EVAR protocols, 7% of TEVAR protocols, and 8% of cEVAR protocols deviated from current ESVS guidelines. Of those respondents deviating from EVAR guidelines, 17% performed DUS at first follow up, followed by 4% who performed AXR and 1% who performed DUS and AXR. Of those deviating from cEVAR protocol, 7% performed DUS and 1% performed DUS and AXR. From the TEVAR protocol, the most common deviations were MRI/MRA (3%), followed by DUS (2%) at first imaging follow up.

DUS is commonly used to confirm the absence of endoleaks and assess the patency and flow in iliac limbs. However, DUS cannot evaluate stent graft overlap, seal integrity, or potential kinking. As a result, it may require an additional CTA or AXR for a comprehensive initial evaluation.^1^^,^^2^^,^^6^ Emerging intra-operative imaging techniques, such as combining angiography with CBCT for thorough assessments during surgery, may reduce the need for post-operative CTA. The prognostic value of the first post-operative CTA and assessment of adequate seal (>10 mm proximally and distally) in predicting late EVAR outcome has been established in several studies.^7^, ^8^, ^9^ Alternatively, AXR employed in combination with DUS before discharge in addition to intra-operative angiography shows high sensitivity and specificity at detecting post-operative complications.^10^ Moreover, sac shrinkage during follow up indicates successful exclusion of the aneurysm from arterial pressure and has been shown to be a predictor of a low risk of EVAR failure during the first five post-operative years.^11^^,^^12^ Early imaging may help the stratification of patients at risk of late failure after EVAR.^7^, ^8^, ^9^^,^^13^

cEVAR procedures, such as fenestrated or branched EVAR, may require individualised follow up protocols tailored to the device, type of repair, and risk of late failure. Data suggest that fenestrated and branched EVAR for complex AAA have acceptable outcomes and complication rates,^14^, ^15^, ^16^ but the re-intervention rates range 24–39%.^14^^,^^16^^,^^17^ The most common complication of cEVAR is target vessel instability, mainly caused by type 1c or type 3c endoleaks or occlusions, which requires secondary endovascular or surgical intervention. These relatively high complication rates and increased risk of secondary re-intervention may necessitate close, lifelong surveillance after cEVAR. This surveillance should include evaluation of target vessel patency and integrity next to measurement of the aortic aneurysm sac size and endoleak assessment. CTA has traditionally been the primary imaging modality for follow up after cEVAR, but this survey points out that DUS, potentially enhanced with contrast, is used as often. However, the literature on the accuracy of DUS in follow up after cEVAR remains limited, and larger studies with longer follow up are needed.^18^, ^19^, ^20^, ^21^, ^22^

After TEVAR, long term complications may also occur, including stent graft migration and progressive dilatation of untreated parts of the aorta with a risk of re-interventions.^23^, ^24^, ^25^ DUS is not suited for imaging of the thoracic aorta and CT is generally the preferred imaging modality. Although not frequently used by the respondents, MRI could serve as an alternative to CT. It eliminates the need for ionising radiation and iodinated intravenous contrast. However, MRI has several drawbacks, including higher costs, lower resolution, longer acquisition times, and limited ability to visualise metallic stent graft components and surrounding structures. MRI may have an increasingly important role, particularly for younger patients and those with decreased renal function, for whom the long term effects of contrast induced nephropathy and cumulative radiation exposure should be considered. MRI techniques are evolving and may have a greater role in the future.^26^

Nearly all respondents reported having a standardised protocol for all or most patients. However, the risk of long term complications is different for some patients, and pre- and post-operative risk factors have been identified. Patients undergoing EVAR outside the manufacturer's IFU, or with a proximal aortic neck diameter of ≥30 mm, angulation of ≥60°, and an iliac artery diameter of ≥20 mm are at increased risk of late complications. Similarly, patients with endoleaks, or stent overlap or seal of <10 mm on post-operative CTA have an increased risk of late complications.^1^ Fortunately, the majority of patient do not have these risk factors and are thus at low risk of late complications. Most follow up imaging in asymptomatic patients does not uncover significant issues or lead to re-intervention. However, regular prophylactic imaging incurs considerable costs, with implications for the overall lifetime expense of EVAR and raises concerns about its cost effectiveness in health economic evaluations. Beside the burden for patients and the overall costs of the intervention, observational data have shown no survival advantage for patients with complete follow up *vs*. patients with incomplete follow up after EVAR.^27^^,^^28^ Despite this, most survey respondents indicated that they continue EVAR follow up imaging lifelong (51%), the remainder continuing surveillance until a certain age of the patient or a certain number of years has been reached.

The fact remains that surveillance frequency following EVAR is supported by little evidence.^29^ Current ESVS guidelines on follow up after EVAR state three recommendations, none of them level A evidence and none detailing the length or intensity of follow up.^1^ This is reflected in the current results, which show considerable variety in imaging follow up protocols. Moreover, this study shows that many centres adhere to standardised protocols with frequent follow up imaging, as has been common practice since the advent of EVAR. High quality studies investigating follow up, resulting in evidence based strong recommendations are therefore needed.

Frequent CTA imaging after EVAR is problematic due to the associated costs, cumulative radiation exposure, and nephrotoxicity of iodinated contrast. Therefore, further patient stratification and reduction of unnecessary EVAR follow up imaging is desirable.**^20^**

### Limitations

This worldwide survey had some limitations. Firstly, the data may have been incomplete due to respondents not providing comprehensive information on their follow up protocols. However, in providing an overview of worldwide follow up practices for endovascular aneurysm repair, this may stir the debate and be an incentive to initiate follow up studies investigating optimal follow up imaging and timing. Secondly, the unequal representation of various continents and medical specialties constrains the generalisability of the findings. This may have led to over representation of European countries (64% of participants), where imaging such as CTA is more accessible and where EVAR and corresponding follow up regimens are more established in vascular care. Future prospective research should demonstrate the safety of minimal follow up for the first five years after successful EVAR with low chance of complications. Moreover, it should aim to include a more diverse and representative cohort to better capture global practices (including differences between high, medium, and low income countries or private *vs*. public healthcare systems) and facilitate the development of universally applicable guidelines.

### Conclusion

This global survey shows substantial variation between follow up imaging protocols after EVAR, cEVAR, and TEVAR. Most of these protocols lead to long term radiation exposure during follow up. There is a pressing need to identify optimal individual and procedural specific follow up after endovascular aneurysm repair.

## Funding

None.

## CONFLICTS OF INTEREST

None.

## APPENDIX A

European Vascular Research Collaborative (EVRC)

Alexandra Gratl^a^, Florian Enzmann^a^, Gert J. de Borst^b^, Lewis Meecham^c^, Stefano Ancetti^d^, Paolo Spath^d^, Albert Busch^e^, Bergrós Jóhannesdóttir^6^, Alexander Gombert^g^, Mohammad E. Barbati^g^, Panagiotis Doukas^g^, Katariina Noronen^f^, Robert Hinchliffe^i^, Alexandru Predenciuc^j^, Qasam Ghulam^k^, Angelos Karelis^l^, Maram Darwish^m^, Cecilie Markvard Møller^n^, Matt Spreadbury^o^, Willemien van de Water^p^, Desiree van den Hondel^q^, Alexander Croo^r^, Gilles Uijtterhaegen^s^, Ryan Gouveia e Melo^t^, Vaiva Dabravolskaite^u^, Christian Zielasek^u^, Salome Weiss^u^, Vishal Amlani^v^, Aoife Kiernan^w^, Petar Zlatanovic^x^, Nikolaos Patelis^y^, Max Hoebink^z^, Harm Ebben^z^, Kak Khee Yeung^z^

^a^Department of Vascular Surgery, Medical University of Innsbruck, Innsbruck, Austria.

^b^Department of Vascular Surgery, University Medical Center Utrecht, Netherlands.

^c^Department of Vascular Surgery, University Hospital Wales, Cardiff, UK.

^d^Department of Vascular Surgery, Bologna University Hospital, Bologna, Italy.

^e^Division of Vascular and Endovascular Surgery, Department of Visceral, Thoracic and Vascular Surgery, Technical University of Dresden and University Hospital Carl-Gustav Carus, Dresden, Germany.

^f^Department of Vascular Surgery, Bordeaux University Hospital, Bordeaux, France.

Clinic of Vascular and Endovascular Surgery, RWTH Aachen University Hospital, Aachen, Germany.

^g^Department of Vascular Surgery, Helsinki University Hospital, Helsinki, Finland.

^h^Department of Vascular Surgery, Bristol Medical School, University of Bristol, United Kingdom.

^i^Division of Vascular Surgery, Institute of Emergency Medicine, Nicolae Testemitanu State University of Medicine and Pharmacy, Chisinau, Moldova.

^j^Department of Vascular Surgery, Capio Privathospital, Hellerup, Denmark.

^k^Vascular Center, Department of Thoracic Surgery and Vascular Diseases, Skåne University Hospital, Malmö, Sweden.

^l^Health Education England, Vascular and Endovascular Surgery, East Midlands Deanery, Nottingham, United Kingdom.

^m^Department of Cardiothoracic and Vascular Surgery, Aarhus University Hospital, Denmark.

^n^Department of Vascular Surgery, Oslo University Hospital, Oslo, Norway.

^o^Department of Vascular Surgery, Maastricht University Medical Center, Maastricht, the Netherlands.

^p^Department of Vascular Surgery, Frisius MC, Leeuwarden, the Netherlands.

^q^Department of Vascular Surgery, AZORG Hospital, Aalst, Belgium.

^r^Department of Vascular Surgery, AZ Sint-Lucas, Brugge, Belgium.

^s^Vascular Surgery Department, Hospital Santa Maria, Unidade Local Saúde de Santa Maria;

^t^Faculdade de Medicina da Universidade de Lisboa; Centro Cardiovascular da Universidade de Lisboa (CCUL@RISE).

^u^Department of Vascular Surgery, Inselspital, Bern University Hospital, Bern, Switzerland.

^v^Department of Vascular Surgery, Sahlgrenska University Hospital, Gothenburg, Sweden.

^w^Department of Vascular Surgery, Bon Secours Health System, Dublin, Ireland.

^x^Clinic for Vascular and Endovascular Surgery, University Clinical Centre of Serbia, Belgrade, Serbia.

^y^Department of Vascular Surgery, Mouwasat Hospital, Saudi Arabia.

^z^Department of Surgery, Amsterdam University Medical Center, Amsterdam, The Netherlands.
