# Supplementary material for: Follow up Imaging Protocols after Endovascular Aneurysm Repair: Results of the International FOREVAR Survey
Source: EJVES Vasc Forum. 2025 Jul 28;64:190–6. doi: 10.1016/j.ejvsvf.2025.07.005 (PMC12799499; doi:10.1016/j.ejvsvf.2025.07.005)
Supplement: Multimedia component 1 [file mmc1.docx]

**Supplementary material**

**S1. The complete FOREVAR Survey**

| **Questions in the FOREVAR-survey** |
| --- |
| Q1. By filling in this survey you agree to: Processing the data you have provided. Thereby, saving and analyzing your answers for research purposes only, all data will be handled by the responsible researcher(s) only and will not be shared without permission.  Your answers being processed anonymously.  I want to continue with the survey: |
| Q2. If necessary, do you give us permission to contact you via e-mail, to answer any questions regarding your given answers? |
| Q3. Please enter your information below: |
| Q4. In which country is your practice located? |
| Q5. What is your specialty? |
| Q6. What kind of practice are you working at? |
| Q7. Please enter your information |
| Q8. In which country is your practice located? |
| Q9. What is your specialty? |
| Q10. Is there a standardized* imaging follow-up protocol in your practice after endovascular aneurysm repair? *Given that no complications (i.e. endoleaks) occurred related to the procedure. |
| Q11. Do you have a standardized follow-up imaging protocol for all patients, and all vascular surgeons, in your practice for the standard elective EVAR? |
| Q12. What is your standard follow-up imaging protocol for patients after standard elective EVAR before discharge?(Multiple answers possible) |
| Q13. What type of imaging is used after the standard elective EVAR in the first imaging follow-up? (Multiple answers possible) |
| Q14. How many weeks after the standard elective EVAR is the first imaging follow-up? |
| Q15. If the first imaging follow-up is within IFU and no type 2 EL is detected, will there still be a next standard follow-up in your practice? |
| Q16. How many weeks after the standard elective EVAR is the second imaging follow-up?* |
| Q17. What type of imaging is used in the second imaging follow-up? |
| Q18. Is there any further follow-up in the standard imaging-protocol? |
| Q19. When is the third follow-up, if within IFU, and no type 2 endoleak (EL) is detected, after the standard elective EVAR?**Please specify in weeks |
| Q20. What type of imaging is used during the third follow-up after the standard elective EVAR? (Multiple answers possible) |
| Q21. Where does the follow-up take place? |
| Q22. When does the follow-up end? |
| Q23. After how many years does the follow-up end? |
| Q24. After what age does the follow-up end? |
| Q25. Do you perform complex EVAR (cEVAR) (FEVAR/ BEVAR/chimneys) in your center? |
| Q26. Is your standard protocol for follow-up imaging after cEVAR (FEVAR/ BEVAR/ chimneys) the same as for the standard elective EVAR? |
| Q27. Do you have a standardized follow-up imaging protocol for all patients, and all vascular surgeons, in your practice for the cEVAR? |
| Q28. What is your standard follow-up imaging protocol for patients after cEVAR before discharge? (Multiple answers possible) |
| Q29. How many weeks after the cEVAR is the first imaging follow-up? |
| Q30. What type of imaging is used after the cEVAR in the first imaging follow-up? (Multiple answers possible) |
| Q31. If the first imaging follow-up is within IFU and no type 2 EL is detected, will there still be a next standard follow-up in your practice? |
| Q32. When is the second follow-up, if within IFU, and no type 2 endoleak (EL) is detected, after the cEVAR? **Please specify in months |
| Q33. What type of imaging is used in the next follow-up? |
| Q34. Is there any further follow-up in the standard imaging-protocol after cEVAR? |
| Q35. When is the third follow-up, if within IFU, and no type 2 endoleak (EL) is detected, after the cEVAR? **Please specify in months |
| Q36. What type of imaging is used during the further follow-up after the cEVAR? (Multiple answers possible) |
| Q37. When does the follow-up end? |
| Q38. After how many years does the follow-up end? |
| Q39. After what age does the follow-up end? |
| Q40. Do you perform TEVAR in your center? |
| Q41. Is your standard protocol for follow-up imaging after TEVAR the same as for the standard elective EVAR? |
| Q42. Do you have a standardized follow-up imaging protocol for all patients, and all vascular surgeons, in your practice for TEVAR? |
| Q43. What is your standard follow-up imaging protocol for patients after TEVAR before discharge? (Multiple answers possible) |
| Q44. How many weeks after the TEVAR is the first imaging follow-up? |
| Q45. What type of imaging is used after the TEVAR in the first imaging follow-up? (Multiple answers possible) |
| Q46. If the first imaging follow-up is within IFU and no type 2 EL is detected, will there still be a next standard follow-up in your practice? |
| Q47. When is the second follow-up, if within IFU, and no type 2 endoleak (EL) is detected, after TEVAR? **Please specify in months |
| Q48. What type of imaging is used in the next follow-up after TEVAR? |
| Q49. Is there any further follow-up in the standard imaging-protocol for TEVAR? |
| Q50. When is the third follow-up, if within IFU, and no type 2 endoleak (EL) is detected, after TEVAR? **Please specify in months |
| Q51. What type of imaging is used during the further follow-up after TEVAR? (Multiple answers possible) |
| Q52. Where does the follow-up take place? |
| Q53. When does the follow-up end? |
| Q54. After how many years does the follow-up end? |
| Q55. After what age does the follow-up end? |

*Abbreviations: Endovascular aortic repair (EVAR); Instructions for use (IFU); Complex EVAR (cEVAR); Thoracic EVAR (TEVAR); Endoleak (EL).*
